# A Multimodal Stimulation Cell Culture Bioreactor for Tissue Engineering: A Numerical Modelling Approach

**DOI:** 10.3390/polym12040940

**Published:** 2020-04-18

**Authors:** João Meneses, João C. Silva, Sofia R. Fernandes, Abhishek Datta, Frederico Castelo Ferreira, Carla Moura, Sandra Amado, Nuno Alves, Paula Pascoal-Faria

**Affiliations:** 1 Centre for Rapid and Sustainable Product Development (CDRSP-IPLeiria), 2430-028 Marinha Grande, Portugal; 2 Instituto de Biofísica e Engenharia Biomédica, Faculdade de Ciências, Universidade de Lisboa, 1749-016 Lisboa, Portugal; 3 Department of Bioengineering and iBB-Institute for Bioengineering and Biosciences, Instituto Superior Técnico, Universidade de Lisboa, Av. Rovisco Pais, 1049-001 Lisbon, Portugal; 4 Soterix Medical, Inc., New York, NY 10001, USA; 5 Department of Biomedical Engineering, City College of New York, New York, NY 10031, USA

**Keywords:** cylindrical perfusion bioreactor, multimodal stimulation, cytotoxicity study, material characterization, bone tissue engineering, finite element analysis, electrical stimulation

## Abstract

The use of digital twins in tissue engineering (TE) applications is of paramount importance to reduce the number of in vitro and in vivo tests. To pursue this aim, a novel multimodal bioreactor is developed, combining 3D design with numerical stimulation. This approach will facilitate the reproducibility between studies and the platforms optimisation (physical and digital) to enhance TE. The new bioreactor was specifically designed to be additive manufactured, which could not be reproduced with conventional techniques. Specifically, the design suggested allows the application of dual stimulation (electrical and mechanical) of a scaffold cell culture. For the selection of the most appropriate material for bioreactor manufacturing several materials were assessed for their cytotoxicity. Numerical modelling methods were then applied to the new bioreactor using one of the most appropriate material (Polyethylene Terephthalate Glycol-modified (PETG)) to find the optimal stimulation input parameters for bone TE based on two reported in vitro studies.

## 1. Introduction

Tissue Engineering (TE) approaches include the culture of stem cell seeded on a scaffold for cell growth and differentiation in an environment that mimics the native tissue. In order to increase cell rate and time of survival, the culture medium has to be periodically replaced to supply nutrient and growth factors to cells and removal of toxic cell by-products from the culture to avoid cell necrosis and limit extracellular matrix formation [1]. This can be done by using dynamic culture conditions such as a perfusion flow bioreactor that facilitates mass transport and waste removal to/from the cells. Perfusion bioreactors can also be used to apply mechanical stimulation via fluid flow shear stress (FFSS) and/or hydrostatic pressure by modulating the bioreactor inlet and outlet flow rate [2]. In addition to the perfusion system, an electric field (E-field) stimulation apparatus can be added to the perfusion bioreactor design without compromising the flow. In vitro studies show that electrical stimulation promotes cell proliferation, matrix development, maturation and cell differentiation [3].

The continuous innovations in additive manufacturing (AM) allow the building of more complex 3D shapes and structures with ease, providing extensive benefits over traditional manufacturing technologies. AM technologies are gradually becoming more popular and ubiquitous due to its capability of manufacturing highly customised parts at lesser cost and time [4]. AM technologies are becoming rapidly available to cell culture laboratories worldwide and they can be used to produce specialised complex bioreactors for TE, allowing researchers to share their experimental designs and comparing the results obtained for common cell culture platforms. To this end, some challenges can be overcome by selecting appropriate available AM technologies. Additionally, there are several manufacturing parameters that should be considered, for instance, nozzle diameter, bed and chamber temperatures, extrusion velocity, different postprocessing methods or availability of 3D printable materials. The combination of these multiple factors gives rise to different surface characteristics and mechanical properties that will introduce variability in the conditions provided by the cell culture bioreactor. Other challenges to overcome are bioreactor design constraints imposed by AM technology, which must be taken into account for a successful production and to reach a design reproducibility (e.g., layer thickness, geometry angle constraints and support material) [5,6,7].

This study proposes a novel cylindrical bioreactor design that includes an integrated perfusion system, requiring a AM production approach. Cytotoxicity tests were performed for several 3D printable materials after production and postprocessing (when required) in order to find the most suitable material for producing a bone cell culture bioreactor. Some of these materials have already been used in different cell culture scaffolds in in vitro studies, thus it is expected that such materials are biologically able to be used for bioreactor fabrication. This new design of bioreactor is an update from a previous version presented by our group [8]. This new design allows the simultaneous application of E-Field stimulation and fluid flow mechanical stimulation. To investigate the new design, and its capabilities of applying adequate stimuli, the results from a numerical finite element analysis (FEA) performed on the new bioreactor model are presented. Electrical and mechanical stimulation conditions in the region-of-interest (ROI) were considered for bone cell stimulation optimisation, according with reference values obtained from two previous in vitro studies on bone cell stimulation, one applying mechanical stimulation [9], and the other using E-Field stimulation [10].

## 2. Materials and Methods

### 2.1. Multimodal Stimulation Bioreactor Design

**Figure 1:**
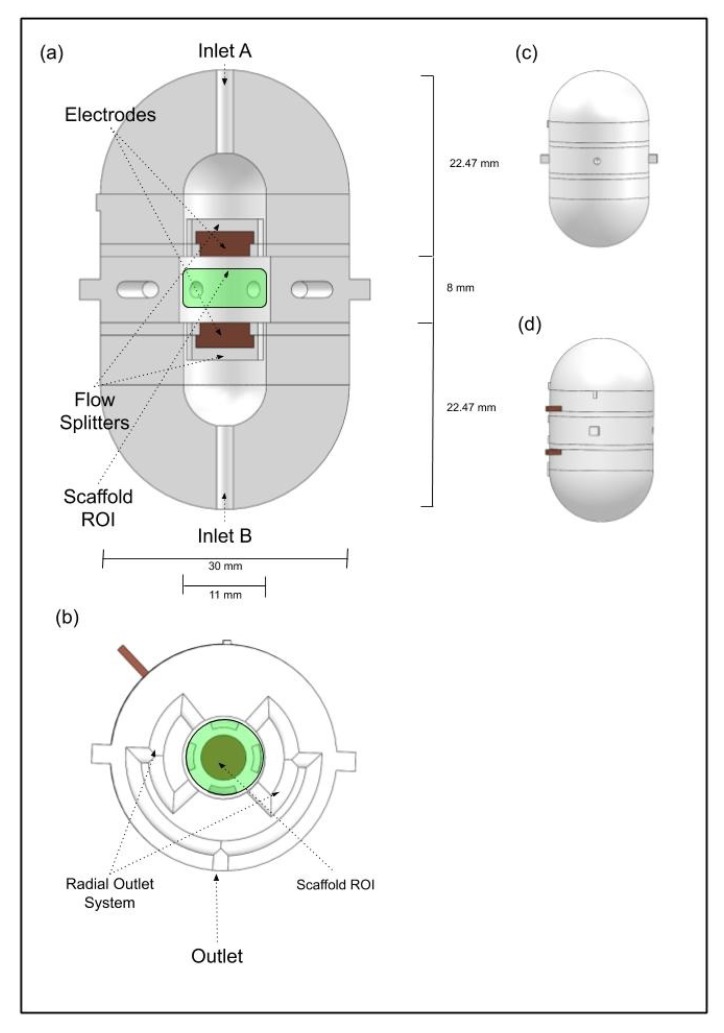
Novel bioreactor design: (**a**) Vertical cut view of the bioreactor design, where the parallel electrodes set up, the upper and bottom inlets and the inlet flow splitters can be observed. (**b**) Horizontal cut view of the bioreactor design, where the radial outlet system can be observed. The green regions represent the region-of-interest (ROI) where the scaffold will be placed, represented by a cylinder with 4 mm of height and a diameter of 10 mm. (**c**) CAD bioreactor design assembled in frontal view, the main outlet hole is visible in the middle. (**d**) CAD bioreactor design assembled in lateral view, showing both electrode connector wires (in brown).

The cylindrical perfusion bioreactor was developed with the purpose of achieving a uniform fluid flow in the region where the scaffold will be positioned (bioreactor centre). The bioreactor design is presented in Figure 1. It contains two symmetric inlets opposed 180° from each other, and four outlets disposed radially to the scaffold (Figure 1a,b). To prevent the inlet direct flow collision with the scaffold, one flow splitter was added between each inlet and the scaffold to establish indirect flow prevalence in the scaffold region (Figure 1a). The radial outlet system was designed to ensure that the fluid exiting from every outlet branch converges into a single outlet going through the exact same distance, and thus keeping the fluid pressure drop homogeneous among all four outlet branches (Figure 1b). Each inlet and outlet has a hose joiner for connection with the perfusion pump system. The cell culture chamber is divided into two parts to allow the placement and removal of the cell culture scaffold and cell seeding. The top part acts as a lid that contains an extruded thread and the bottom part contains the complementary cut thread. The scaffold is kept in place by the top and bottom parts.

To couple an electrical stimulation apparatus to the designed perfusion bioreactor, electrodes were embedded in the back part of each fluid flow splitter. The surface of each electrode in contact with the interior part of the bioreactor is a circle with a diameter of 6 mm made of high-graded steel, and the two electrodes surfaces are separated by 8 mm (Figure 1a). This parallel plate set-up was compared with other common electrical stimulation set-ups in our previous study [11]: the parallel plate capacitor geometry was selected for our design because it results in an E-field distribution with uniform magnitude and radially oriented between the electrodes while other studied set-ups originated E-fields with different directions and magnitudes for the same region-of-interest (ROI) [11]. The perfusion bioreactor was designed using SOLIDWORKS 2018 Student Edition (Dassault Sistèmes) and exported to the STEP format, which is compatible with the COMSOL Multiphysics software used for FEA.

### 2.2. Material Samples Production

Simple parallelepiped samples with dimensions (depth: 10 mm, width: 10 mm, height: 5 mm) were drawn in SOLIDWORKS 2018 Student Edition (Dassault Sistèmes), then exported to the stereolithography file format (*.stl). This file was then imported to each AM technology native software as described in Table 1. All samples from the same material were printed under the same AM conditions. Table 1 summaries the type and AM methodology for each material sample obtained. Materials used were selected based on AM-compatible materials, suitable mechanical properties, cost, easy processing and material transparency. The latter feature plays a key role for real-time visualisation of the scaffold during in vitro bioreactor cultures.

### 2.3. In Vitro Cytotoxicity Tests

The biocompatibility of the different materials considered for the bioreactor fabrication was assessed using L929 mouse fibroblasts (ATCC number CCL-1) and following the ISO 10993-5 and ISO 10993-12 guidelines [12]. Prior to the test, the materials were sterilised by ultraviolet (UV) exposure overnight, ethanol 70% washing and incubation with a 1% antibiotic–antimycotic (Anti–Anti, Gibco™, Fisher Scientific, Waltham, MA, USA) solution in phosphate-buffered saline (PBS, Gibco™, Fisher Scientific, USA). All materials were evaluated by performing the indirect extract test and direct contact test. L929 fibroblasts were cultured on tissue culture polystyrene (TCPS) plates with Dulbecco’s Modified Eagle’s Medium (DMEM, Gibco™, Fisher Scientific, USA) supplemented with 10% (*v*/*v*) Fetal Bovine Serum (FBS, LifeTechnologies, USA) and with 1% Anti–Anti in an incubator at 37 °C/5% CO_2_, to be used as negative control. Latex was used as positive control for cell death. Extracts were prepared by incubating the materials in DMEM + 10%FBS + 1% Anti–Anti culture media at a ratio of 0.2 g of material/mL for 72 h at 37 °C/5% CO_2_. This ratio ensures that test sample covers on tenth of the cell layer surface, acording to the ISO 10993-5:2009 [12]. L929 fibroblasts were seeded on TCPS plates at a cell density of 10^5^ cells per well and cultured for 24 h at 37 °C/5% CO_2_ to generate a confluent monolayer. For the indirect extract test, the culture media was removed and L929 cells were exposed to the material extract’s conditioned medium for 72 h at 37 °C/5% CO_2_. Then, extract conditioned media were removed and the MTT (3-(4,5-dimethylthiazol-2-yl)-2-5 diphenyl tetrazolium bromide) assay (In Vitro Toxicology Assay Kit MTT based, Sigma-Aldrich, St. Louis, MO, USA) was performed in accordance with the manufacturer’s guidelines. Briefly, cells were incubated with MTT solution (1 mg/mL, yellow) for 2 h at 37 °C, and afterwards the violet formazan product resultant from the MTT metabolic reduction by metabolically active cells was dissolved under agitation using a 0.1 N HCl solution in anhydrous isopropanol (Sigma-Aldrich). Absorbance values of the resultant solutions were measured in a plate reader (Infinite M200 PRO, TECAN, Mannedorf, Switzerland) at 570 nm. The direct contact assay was performed in accordance with the ISO standards mentioned above, individual specimens of the test samples were carefully placed over previously formed confluent monolayer of L929 fibroblasts (in the centre of each of the replicate wells; three replicates were used for each sample). The materials were in direct contact with the L929 cell monolayer, incubated for 72 h at 37 °C/5% CO_2_, according to the ISO 10993-5:2009 standard. Afterwards, cell viability and morphology were evaluated qualitatively under an inverted optical microscope (LEICA DMI3000B, Leica Microsystems, Wetzlar, Germany) equipped with a digital camera (Nikon DXM1200F, Nikon Instruments Inc., Melville, NY, USA) to assess any cytotoxic responses such as the occurrence of halo inhibition effect or abnormal cell morphology.

### 2.4. Multimodal Stimulation

Finite element method analysis was performed using the AC/DC and CFD modules from COMSOL Multiphysics software (version 5.2a, www.comsol.com, Stockholm, Sweden). The *Electric Current (ec)* and *Laminar Flow (spf)* physics interfaces were selected, considering a stationary study. Our goal was to find which input stimulation values originate optimal experimental electrical and flow stimulation conditions according with the experimental studies performed by Mobini et al. [10] and Zhao et al. [9].

Mobini et al. [10] applied electrical stimulation with L-shape electrodes that are immersed in the culture chamber, the cell culture takes place in the region between the two electrodes. Using an input voltage of 2.2 V direct current (DC) resulted in the delivery of an E-Field of 100 mV/mm (100 V/m). The induced E-field magnitudes reported produced a 3-fold increase in the rate of osteogenic differentiation of mesenchymal stem cells in comparison with non-stimulated cells. Zhao et al. [9] work found that the optimal flow rates, under which the highest fraction of scaffold surface area is subjected to a wall shear stress that induces mineralisation, are mainly dependent on the scaffold geometries. Nevertheless, the variation range of such optimal flow rates are within 0.5 to 5 mL/min (in terms of fluid velocity: 0.166–1.66 mm/s), considering different scaffold geometries and according to a mechano-regulation theory where extracellular matrix mineralisation would be stimulated when wall shear stress was in certain described ranges [9].

**Figure 2:**
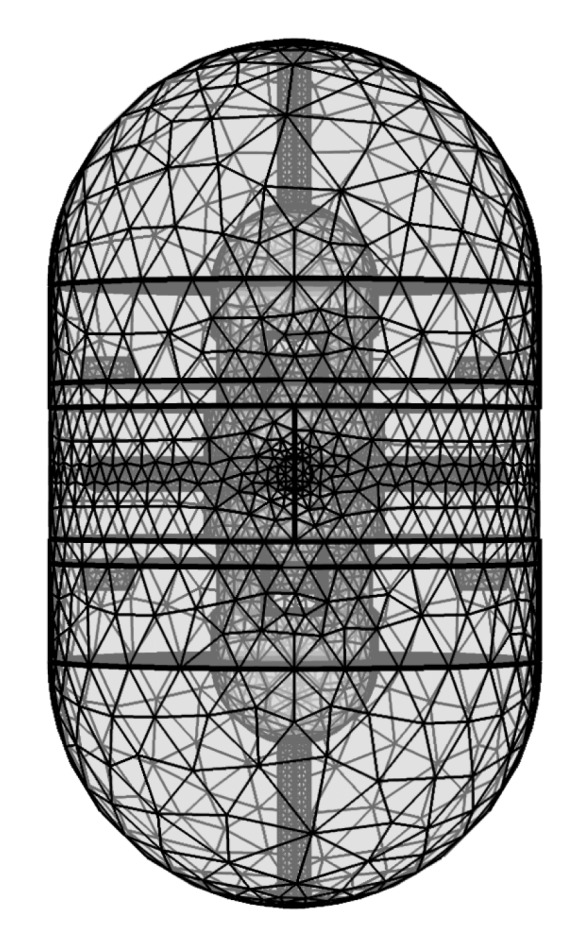
Bioreactor geometry volume mesh created using COMSOL Multiphysics, with 1.9 × 10^6^ elements, and an average element quality of 0.65.

The CAD model of the bioreactor was imported into COMSOL where a physics-controlled mesh was generated with 1.9 × 10^6^ tetrahedral volume elements, and an average element quality of 0.65, as shown in Figure 2. The final geometry was composed of four distinct domains: one fluidic domain, made from culture medium material with electrical conductivity of 1.5 S/m and relative electrical permittivity of 80.1 [13]; one construction domain, consisting in PETG material with electrical conductivity 10^−14^ S/m (resistivity 10^12^ Ohm/cm) and relative electrical permittivity of 2.5 [14]; and two electrode domains, made from Steel AISI 4340 material with electrical conductivity of 4.032 × 10^6^ S/m and relative electrical permittivity of 1. The temperature for this simulation was set at 37 °C.

For the COMSOL laminar flow study, the fluidid domain representing the culture medium was assumed as an homogeneous and incompressible Newtonian fluid with a volume density of 1000 kg/m^3^ and dynamic viscosity of 8.1 × 10^−4^ Pa ·s. Considering that the ROI diameter is 0.010 m, and the fluid velocity in the same region is 0.0016 m/s [9], the calculated Reynolds number was 18.51, which is less than the 2300 turbulent threshold [15]), and a single-phase laminar flow regime was also considered. Physics model solver applies the incompressible form of the Navier–Stokes and continuity equations. Boundary conditions were set for all reactor’s walls with a no-slip condition, the outlet was set at a reference constant pressure of 0 Pa, the two inlets were set at the same value for inflow velocity and the velocity vector field was assumed normal to the inlet surface. To find the inlet velocity value that generates an optimal velocity inside the ROI chamber, we determined the external inlet and outlet conditions that produced the desired values that adequately translate the necessary flow conditions in the cell culture scaffold region. For this laminar flow study, the system initial condition was at rest, so the velocity field and pressure of the entire system was set to zero.

We have also determined the external electrical stimulation required to produce the optimal E-field distribution in the same ROI, according to Mobini et al. [10]. A stationary study was considered in COMSOL, as DC stimulation parameters do not change over time. This study solves the Laplace’s equation (∇·(σ∇ϕ))=0, where ϕ is the electrostatic potential and σ is the electric conductivity, and takes the gradient of the scalar potential to determine the induced E-field. This procedure assumes that the quasi-statics approximation holds [16]. In this approximation, tissues are considered to be purely resistive with no capacitive components, which is valid for DC stimulation (low frequency range) [16]. The following boundary conditions were imposed; an electric insulation condition was added to all external boundaries of the bioreactor walls, continuity of the normal component of the current density in all interior boundaries and electrical potential boundary conditions were added to each electrode interior boundary surfaces relative to the ROI. One electrode boundary surface was set at 0 V potential (ground electrode). The other electrode surface boundary potential was varied in order to find the value that resulted in a predicted E-field in the ROI of 100 mV/mm, according to Mobini et al. [10]. All other domains and boundaries were set at 0V at the beginning of the simulation, to establish an initial resting condition state of the entire system.

## 3. Results and Discussion

### 3.1. Cytotoxicity Study

**Figure 3:**
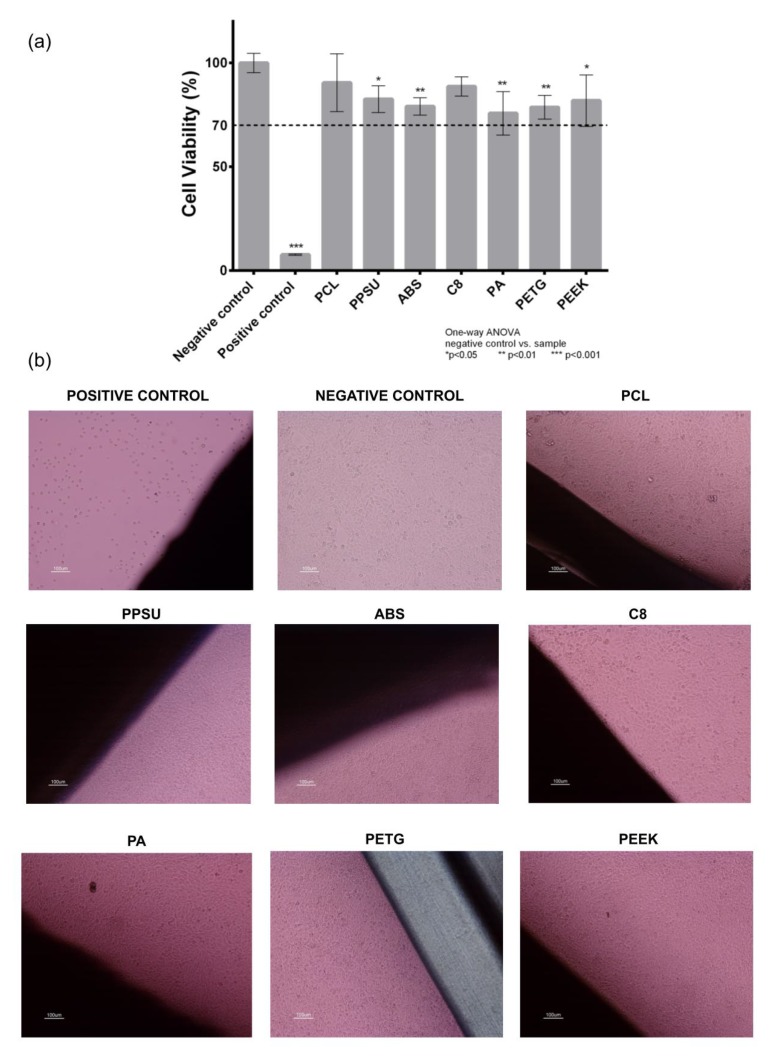
Cytotoxicity assay with L929 mouse fibroblast according to ISO 10993-5 standards: (**a**) indirect contact (MTT protocol); (**b**) direct contact (digital images of the material samples and the negative and positive controls, fresh culture medium and Latex, respectively). A one-way ANOVA with no corrections for multiple comparisons (Fisher’s test) statistical analysis was performed using GraphPad Prism6.

Candidate materials for the fabrication of the bioreactor platform were evaluated in terms of their cytotoxicity effect using a L929 fibroblast cell line and following the ISO 10993-5 standards as described in the Materials and Methods section (Figure 3). Using a one-way ANOVA with no corrections for multiple comparisons (Fisher’s test) it was possible to observe that PCL and C8 materials do not present any response compared to the negative control (*p* > 0.05). Despite the statistically differences visible in Figure 3, ISO standards states that only materials with cell viabilities less than 70% are considered cytotoxic. This way, it is possible to affirm that from all materials tested—PCL (90.48 ± 3.88%), PPSU (82.57 ± 6.45%), ABS (79.05 ± 4.16%), C8 (88.67 ± 4.69%), PETG (78.63 ± 5.69%) and PEEK (81.84 ± 12.39%)—are not cytotoxic materials. The remaining material PA (75.73 ± 10.47%) had some samples with cell viabilities below 70%, and according to the applied criteria it is excluded from the not cytotoxic materials list.

Accordingly, in the direct contact test, cells cultured in contact with all the materials presented normal fibroblast morphology with no evidence of any inhibition halo effect or cell death. According to the cytotoxicity tests results, all candidate materials are suitable for our bioreactor AM fabrication. We will consider C8 and PETG as materials of interest for future design fabrication. C8 is a new material with good layer adhesion and surface quality, which are key features for the perfusion flow. The C8 supplier datasheet reveals that this material has a higher tensile strength than ABS, resulting in improved mechanical characteristics, which are important for the overall robustness of the bioreactor to withstand the tightness of pressure chambers. PETG is a transparent material, which will allow to monitor the interior of the bioreactor in real-time without having to open the bioreactor vessel or interrupt the experiment. PETG also presents good mechanical properties.

### 3.2. Finite Element Analysis Study

Initial conditions for electrical stimulation and laminar perfusion flow were set for FEA, considering the values reported in the following in vitro studies, each showing significant increase in cell proliferation and mineralisation [9,10].

**Figure 4:**
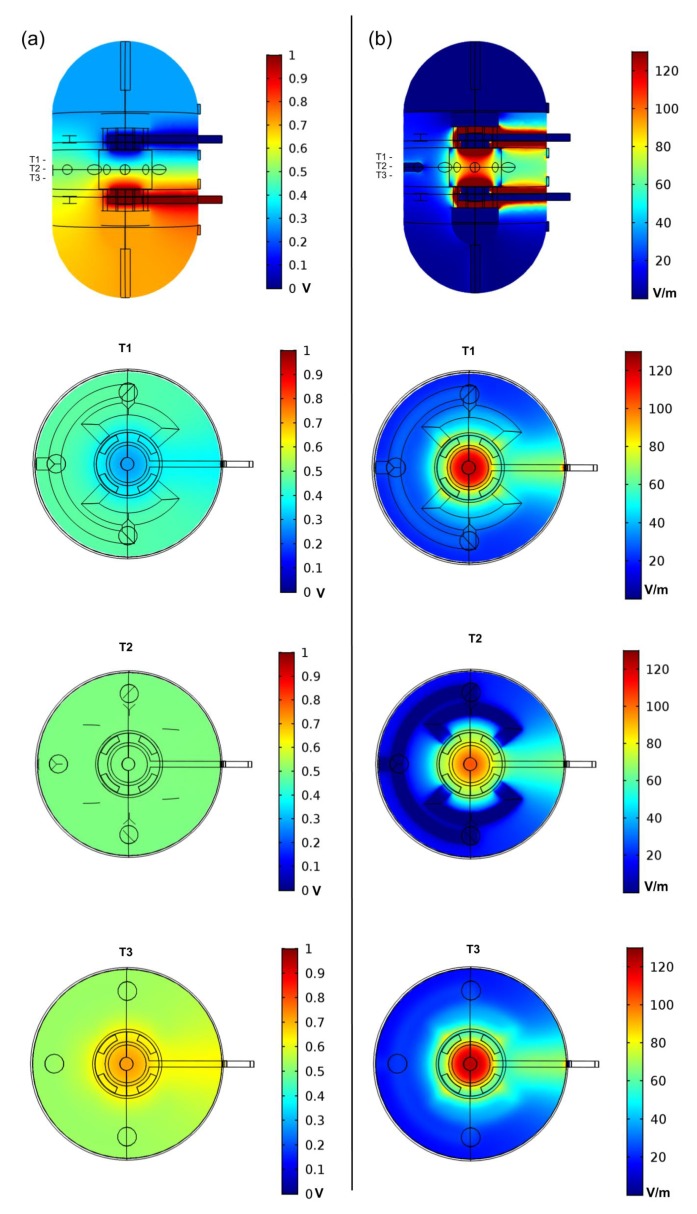
Numerical finite element analysis (FEA) analysis of the proposed bioreactor design with a DC electric stimulation parallel plate capacitor set-up with lateral and top slice views. The three top views represent the ROI upper slice (T1), the ROI middle plane slice (T2) and the ROI bottom slice (T3). (**a**) Electric potential distribution predicted in the bioreactor due to DC stimulation. (**b**) E-Field magnitude distribution predicted for the same electric DC stimulation conditions.

DC stimulation produces a steady-state uniform E-Field, which has been observed to have significant effect on cell differentiation, migration, shape and proliferation [3,10]; therefore, we determined the input electric potential parameters that would reproduce the E-Field magnitude reported by Mobini et al. [10]. The electric potential difference between the electrodes was analytically determined to be 0.8 V, considering a simplified model of a parallel plate electrodes system and an interior culture medium with electrical conductivity and relative permittivity of 1.5 and 1, respectively [17], with an inter-plate distance of 8 mm, and assuming an E-field magnitude between the parallel plates of 100 V/m (100 mV/mm), as reported by Mobini et al. [10]. FEA performed over the volume conductor model of the bioreactor resulted in an E-field below this value, thus the electric potential difference between the plates had to be adjusted to 1.0 V to result in an E-Field average value of 100 V/m in the volume ROI (bottom electrode and upper electrode were set at 1.0 V and 0 V, respectively). With these initial conditions, the E-Field maximum and minimum values predicted in the ROI were 115 V/m and 85 V/m, respectively. Electric potential and E-field distributions in the bioreactor and ROI are presented in Figure 4. The electric potential differences predicted in the ROI are bellow water electrolysis threshold (above 1.229 V at 25 °C [18], Figure 4a). Other studies show that electrical stimulations at lower voltages using input signals of higher frequency (60 kHz) originate bone cell proliferation with lower E-field magnitudes using a capacitively coupled device system (<20 mV/cm). This type of stimulation could also be a possibility to consider for the proposed bioreactor design in future studies [19].

**Figure 5:**
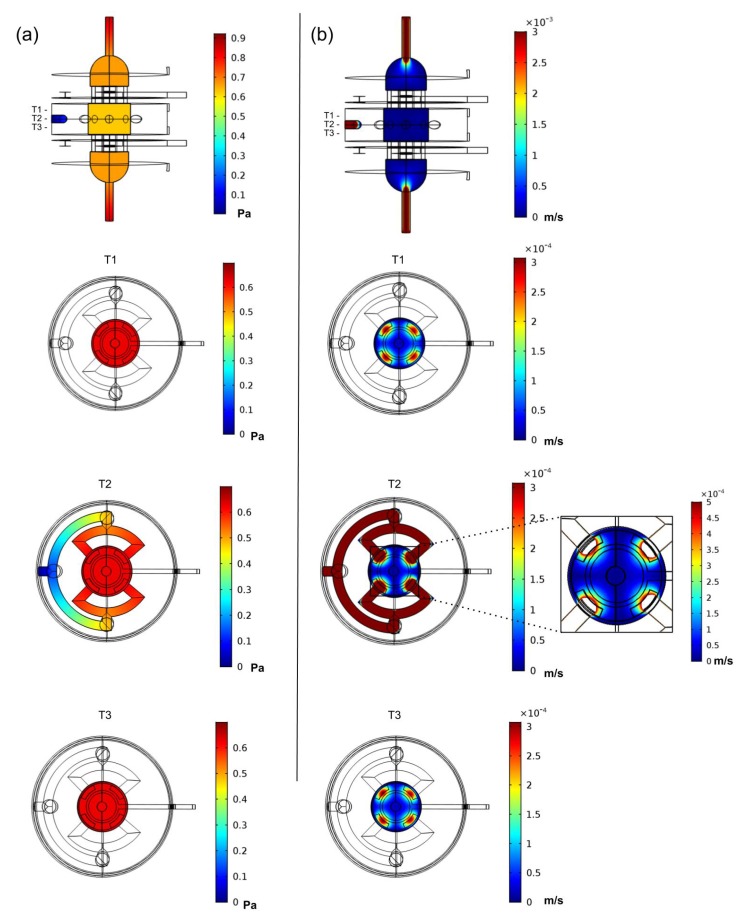
Numerical FEA analysis of the proposed bioreactor design for a laminar perfusion flow with lateral and top slice views. The three top views represent the ROI upper slice (T1), the ROI middle plane slice (T2) and the ROI bottom slice (T3). (**a**) Pressure distribution predicted considering applied inlets velocity of 0.003 m/s and a outlet pressure of 0 Pa. (**b**) Fluid velocity distribution predicted for the same inlet/outlet conditions. The velocity distribution at the ROI middle plane slice is presented in more detail in a top view inset at the right of the slice plane.

Several inlet velocities were tested to find a combination of inlet and outlet flow conditions to originate a flow range in the ROI similar to the one reported in [9]. The conditions determined using this method resulted in a final inlet velocity of 0.003 m/s at each bioreactor inlet, combined with an outlet constant pressure value of 0 Pa. The laminar flow study predicted an average velocity of 1.14 × 10^−4^ m/s, and an average pressure of 0.6 Pa, generating a flow of 2.58 mL/min in the ROI, which is in the considered range of 0.5 to 5 mL/min used for obtaining maximum mineralisation in bone tissue engineering, according to the method in [9]. Velocity and pressure distributions predicted inside the bioreactor model are presented in Figure 5. Despite having predicted adequate fluid velocity and pressure stimulation conditions for the ROI, the introduction of the cell culture scaffold in this ROI will affect the fluid flow; thus, future FEA studies should take into account the scaffold geometry in order to find the appropriate inlet/outlet conditions for each specific scaffold.

The inclusion of a scaffold–cell culture domain may originate local differences in the ROI for the values predicted. The scaffold material and cell culture will have different electrical properties of the culture medium, which may originate local differences in the E-field and electric potential spatial distributions in the ROI. Moreover, the scaffold geometry may affect the fluid flow, thus changing the predicted perfusion inside the scaffold. FEA was essential to predict the adequate input conditions on the designed bioreactor to increase the effectiveness of multimodal cell stimulation in the ROI, considering the experimental conditions determined from the selected in vitro studies [9,10]. Experimental studies using electric or laminar perfusion stimuli often present different stimulation paradigms [3,9]. Parametric sweep numerical studies should be performed to define multiple precise stimulation ranges to apply in experimental protocols, considering the values applied in in vitro studies. Future work would be the validation of the proposed bioreactor model, through AM fabrication using the selected materials from the cytotoxicity assay. Application of different stimuli modalities, such as biochemical induction or electrical stimulation, may induce osteogenic differentiation in human and animal MSCs [10,20,21,22]. Thus, in vitro tests using cell sources with high potential for bone repair, such as osteoblasts or undifferentiated bone marrow-derived mesenchymal stem/stromal cells, will be essential to validate multimodal stimulation ranges predicted by the numerical studies. Water electrolysis and changes in culture medium should be monitored and compared for DC and high frequency stimulation set of parameters. This may lead to a better understanding of the effects that play a critical role when applying combined E-Field and mechanical stimulations to cells and also help to fine-tune experimental protocols for the bioreactor proposed. Considerations on E-field magnitude determined in the experimental study from Mobini et al. [10] shows that the combined approach of design and numerical simulation methods can be used to reproduce values obtained in in vitro experimental studies using electrical stimulation in cell cultures, and replicate these values considering also different cell culture bioreactors. With the methods used in this study, it is possible to obtain an optimised combination of CAD design and numerical modelling methods to engineer a multimodal stimulation bioreactor for stimuli-based tissue engineering applications.

## 4. Conclusions

This study proposes a novel design of a multimodal stimulation bioreactor for TE applications with an experimental validation of candidate materials for AM. This design allows the simultaneous application of fluid perfusion and electrical stimulation to obtain larger outcomes in cell differentiation, migration and proliferation. This study proposes a multimodal stimulation protocol customised for the proposed bioreactor, and it is hypothesised that mesenchymal stem cells differentiation and increased ECM mineralisation may be achieved through the application of this specific stimuli combined application. This study also underlines the importance of combining 3D CAD design and numerical modelling simulations to address the optimal input stimulation conditions, informed from previous experimental studies, to optimise outcomes for cell engineering purposes. A design–numerical modelling approach will be essential to understand the underlying biophysical effects of electric and mechanical stimuli in cell cultures and can be a powerful tool for standardisation of stimulation protocols considering different bioreactor designs and specific TE outcomes.

## Figures and Tables

**Table 1:** Description of sample identity (ID) and material, correspondent supplier and AM methodology.

Sample ID	Material	Supplier	AM Methodology
PCL	Polycaprolactone	FACILAN™ PCL 100, 750 GRAM (MW: 50,000 g/mol), 1.75 MM, 3D4MAKERS, Haarlem, The Netherlands	3DP Technology: FDM; 3D Printer: Creatbot F430; Nozzle Diameter: 0.4 mm; Nozzle Temperature: 165 °C; Heated Bed Temperature: 35 °C; Chamber Temperature: 20 °C; Layer Thickness: 0.02 mm; Printing Speed: 60 mm/s; Infil: 100%.
PA	Polyamide/Nylon	PA Powder, 3D SYSTEMS	3DP Technology: SLS; 3D Printer: 3D System sPro 60 HD-HS; CO_2_ Laser 70W; Layer Thickness: 0.1 mm; Scanning Speed: 6 m/s; Construction Chamber Temperature: 173 °C; Feeding Chamber Temperature: 135°C.
PETG	Polyethylene Terephthalate Glycol-modified	PETG FILAMENT, 750 GRAM, 1.75MM, 3D4MAKERS, Haarlem, The Netherlands	3DP Technology: FDM; 3D Printer: Creatbot F430; Nozzle Diameter: 0.4 mm; Nozzle Temperature: 260 °C; Heated Bed Temperature: 110 °C; Chamber Temperature: 60 °C; Layer Thickness: 0.02 mm; Printing Speed: 60 mm/s; Infil: 100%.
ABS	Acrylonitrile- Butadiene- Styrene	ABS FILAMENT, 750 GRAM, 1.75MM, 3D4MAKERS, Haarlem, The Netherlands	3DP Technology: FDM; 3D Printer: Creatbot F430; Nozzle Diameter: 0.4 mm; Nozzle Temperature: 230 °C; Heated Bed Temperature: 50 °C; Chamber Temperature: 35 °C; Layer Thickness: 0.02 mm; Printing Speed: 60 mm/s; Infil: 100%.
C8	Proprietary Polymer Composite produced by ELOGIOAM 3D MATERIALS	FACILAN™ C8 FILAMENT, 750 GRAM, 1.75MM, 3D4MAKERS, Haarlem, The Netherlands	3DP Technology: FDM; 3D Printer: Creatbot F430; Nozzle Diameter: 0.4 mm; Nozzle Temperature: 195 °C; Heated Bed Temperature: 35 °C; Chamber Temperature: 20 °C; Layer Thickness: 0.02 mm; Printing Speed: 60 mm/s; Infil: 100%.
PPSU	Polyphenylsulfone	PPSU FILAMENT, 500 GRAM, 1.75 MM, 3D4MAKERS, Haarlem, The Netherlands	3DP Technology: FDM; 3D Printer: Creatbot F430; Nozzle Diameter: 0.4 mm; Nozzle Temperature: 380 °C; Heated Bed Temperature: 120 °C; Chamber Temperature: 60 °C; Layer Thickness: 0.02 mm; Printing Speed: 60 mm/s; Infil: 100%.
PEEK	Polyether Ether Ketone	PEEK FILAMENT, 500 GRAM, 1.75 MM, 3D4MAKERS, Haarlem, The Netherlands	3DP Technology: FDM; 3D Printer: Creatbot F430; Nozzle Diameter: 0.4 mm; Nozzle Temperature: 390 °C; Heated Bed Temperature: 120 °C; Chamber Temperature: 60 °C; Layer Thickness: 0.02 mm; Printing Speed: 60 mm/s; Infill: 100%.
